# ^1^H NMR Relaxation Processes in Lung Tissues at Low Magnetic Fields

**DOI:** 10.3390/molecules30194002

**Published:** 2025-10-07

**Authors:** Karol Kołodziejski, Farman Ullah, Łukasz Klepacki, Jerzy Gielecki, Danuta Kruk

**Affiliations:** 1 Department of Physics and Biophysics, University of Warmia and Mazury in Olsztyn, Michała Oczapowskiego 4, 10-719 Olsztyn, Poland; karol.kolodziejski@uwm.edu.pl (K.K.); farman.ullah@uwm.edu.pl (F.U.); 2 Department of Anatomy and Histology, Faculty of Medicine, Collegium Medicum, University of Warmia and Mazury in Olsztyn, 10-082 Olsztyn, Poland; lukasz.klepacki@uwm.edu.pl (Ł.K.); jerzy.gielecki@uwm.edu.pl (J.G.)

**Keywords:** NMR relaxation, tissues, dynamics

## Abstract

Proton spin–lattice and spin–spin NMR relaxation studies were conducted on lung tissue samples from 10 patients. For each case, relaxation properties of tumor tissue were compared with those of the corresponding reference tissue. The spin–lattice relaxation measurements were performed over a wide frequency range, from 10 kHz to 10 MHz, spanning three orders of magnitude. These were complemented by both spin–lattice and spin–spin relaxation data acquired at 18.7 MHz. Notably, the spin–spin relaxation process exhibited a bi-exponential character. This relaxation behavior was quantitatively analyzed using dedicated models to achieve two main goals: to evaluate the diagnostic potential of low-field NMR relaxometry, and to gain insights into the dynamics of water and macromolecules in tissue, in comparison with aqueous solutions of proteins and polymers. The frequency dependence of the spin–lattice relaxation rates was well described by a power-law function, with an exponent of approximately 0.3 closely matching the theoretical prediction for reptation dynamics in polymer systems, associated with the intermolecular relaxation contribution. The combined analysis of spin–lattice and spin–spin relaxation data revealed specific parameters (such as ratios between the relaxation rates or between the amplitudes of individual relaxation components) that can be considered as potential markers of pathological changes affecting molecular dynamics in tissues.

## 1. Introduction

The terms lung cancer or bronchogenic cancer refer to cancers that originate in the lung parenchyma or bronchi [1]. About 90% of cigarette smokers have the highest risk of developing lung cancer. Another risk factor for lung cancer is radiation, which is used to treat breast cancer or non-Hodgkin’s lymphoma, among others [2]. Exposure to metals (nickel, chromium) or aromatic hydrocarbons contributes to the development of lung cancer. Independent of cigarette smoking, idiopathic pulmonary fibrosis also increases the risk of developing lung cancer [3]. The pathophysiology of lung cancer is complex and not fully understood. It has been hypothesized that carcinogens, such as continuous exposure to tobacco smoke, which causes dysplasia of the bronchial epithelium, lead to genetic mutations [4]. The most common genetic mutations responsible for the development of lung cancer are MYC, BCL2 and p53, which are responsible for small-cell lung cancer (SCLC), and EGFR, KRAS and p16 are responsible for non-small-cell lung cancer (NSCLC) [5,6]. Dividing lung cancer into two categories, SCLC and NSCLC, makes it easier to make decisions about the prognosis of the disease and about further treatment [7]. NSCLC is the most common type of lung cancer occurring worldwide [8].

Magnetic Resonance Imaging (MRI) plays a central role in medical diagnostics. MRI exploited differences in quantities referred to as ^1^H relaxation rates (spin–lattice and spin–spin relaxation rates). MRI is performed at a single (high) magnetic field (resonance frequency)—this is related to the need to maintain a high spatial resolution to detect even very small pathological changes. The quantum-mechanical foundation of spin relaxation implies that at high resonance frequencies, the most efficient relaxation mechanism is associated with fast molecular motion [9]. Consequently, the difference in the relaxation rates exploited in MRI primarily stems from alteration of the motion and arrangement of water molecules, caused by pathological changes in tissues [10]. To enquire into slower molecular motion, one needs to perform relaxation experiments at lower resonance frequencies. Profiting from the Fast Field Cycling (FFC) technology, one can perform relaxation experiments over a broad range of resonance frequencies—at least from 10 kHz to 10 MHz (for ^1^H nuclei, this corresponds to the range of magnetic fields from 0.25 mT to 0.25 T). This kind of experiment is referred to as NMR relaxometry. In addition to probing molecular motion on the time scale from ms to a tenth of ns, NMR relaxometry makes it possible to reveal the mechanism of the motion. For instance, one can not only distinguish between translational and rotational motion but also differentiate between isotropic (three-dimensional) translation diffusion and restricted diffusion on surfaces (two-dimensional diffusion) [11,12]. The unique advantages of NMR relaxometry have been greatly appreciated in molecular science, providing insight into the timescale and mechanisms of molecular and ionic motion in a variety of systems, from liquids to solids, including in this range macromolecular systems (proteins, polymers) [13,14,15,16], food products [17,18], nanocomposites and other nanoparticle systems [19]. There are also examples of NMR relaxometry studies of tissues—tissues can be described as complex, heterogeneous systems including macromolecular fractions and water molecules. Thus, it is only natural that, eventually, tissues have also become the subject of NMR relaxometry studies, especially in the context of possible diagnostic applications. Nevertheless, examples of NMR relaxometry studies of tissues are rare. One should point out here the attempt to assess intraoperative margins for breast cancer and reveal the characteristic relaxation features of glioma [20,21,22,23]. NMR relaxometry has also given a unique insight into relationships between the intracellular and extracellular water dynamics in tissues [24,25]. One should also turn attention to the subject of osteoarthritic effects in cartilages [26,27]. The NMR relaxometry studies of sarcoma (authored by some of us) show that pathological changes in tissues affect the slow dynamics of macromolecules and the bound water fraction [28]. Following this line, in our recent work, we have presented a methodology for the analysis of NMR relaxometry data for tissues, indicating a set of characteristic relaxation features (markers), using colon tissues as an example [29]. Eventually, it is important to point out that to exploit the diagnostic potential of frequency-dependent relaxation studies, a prototype scanner combining MRI and FFC has been developed [30].

In this work, we address the subject of relaxation properties of lung tissues, including tumor tissues and healthy tissues. For this purpose, in addition to exploiting NMR relaxometry (performing ^1^H spin–lattice relaxation experiments in the frequency range from 10 kHz to 10 MHz), we have carried out ^1^H spin–lattice and spin–spin relaxation studies at 18.7 MHz. The inclusion of the spin–spin relaxation studies considerably extends the insight into the molecular motion in tissues. This comprehensive approach contributes to the (limited) knowledge about molecular dynamics and arrangement in tissues and carries a diagnostic potential.

## 2. Results and Analysis

^1^H spin–lattice and spin–spin relaxation experiments were conducted on lung cancer tissues. The tissue samples were obtained after surgery, and the experiments started in about 30–40 min after receiving the samples. In case an immediate (after 30–40 min) experiment was not possible, the samples were stored at a low temperature (−24 °C) for a short time (up to 3 h). A total of 2 samples were obtained for patients 1–9 and one sample for patient 10 (this gives 19 samples in total). The sets of two samples (for patients 1–9) include a sample of pathological tissue (tumor) and a sample of tissue obtained from the margin (peri-tumoral). For patient 10, only the sample of pathological tissue was investigated. The histopathological characterization of the tumors is collected in Table 1.

**Figure 1:**
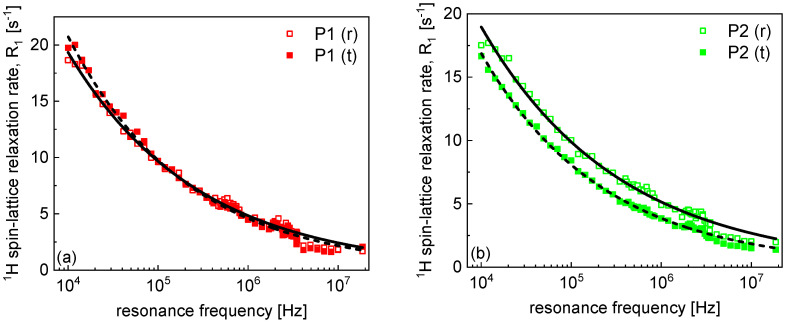

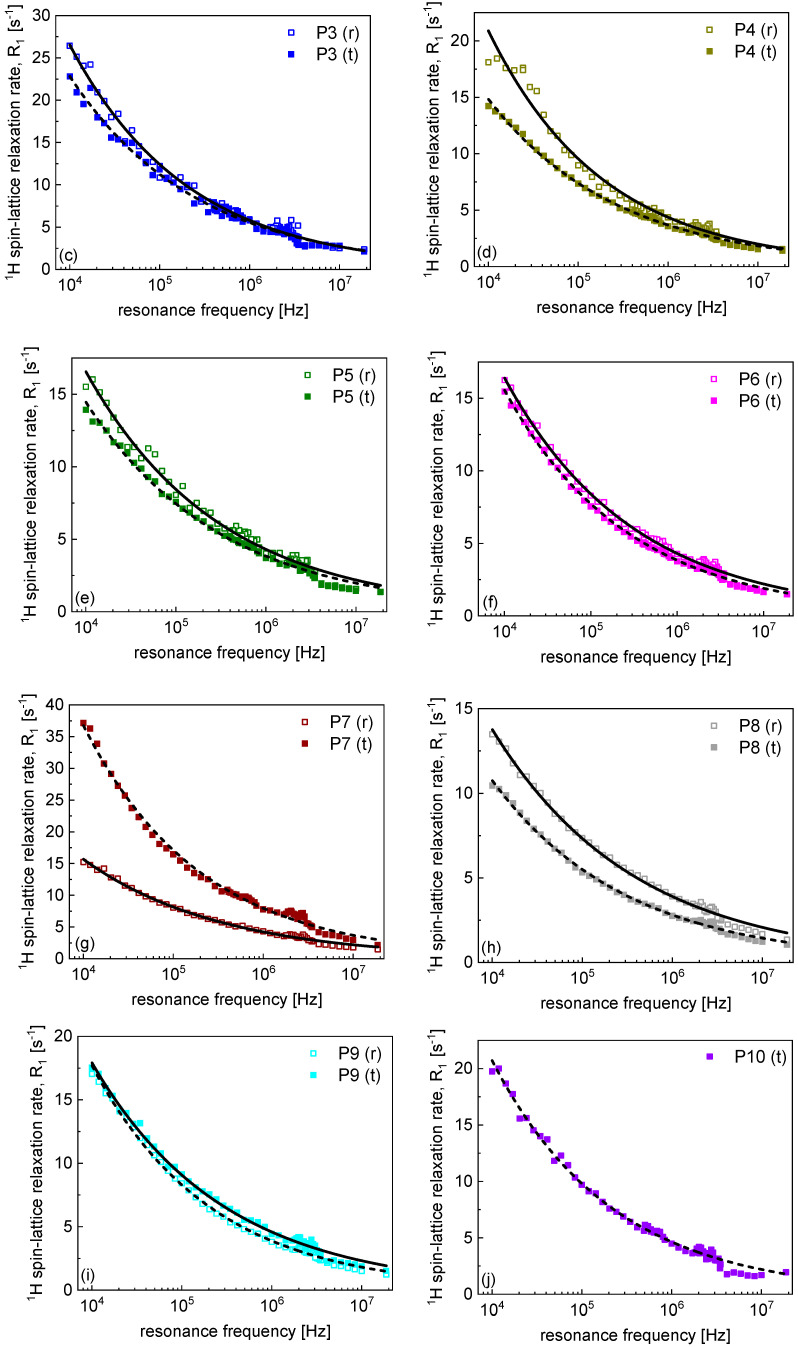
(**a**–**j**) ^1^H spin–lattice relaxation data for lung tissues. Solid lines and dashed lines—fits in terms of Equation (1) for tumor and reference samples, respectively (R-square values for the fits range between 0.993 and 0.997).

The ^1^H spin–lattice relaxation data collected by means of FFC-NMR relaxometry are shown in Figure 1 for the individual patients. The data obtained for the pathological samples are denoted as open symbols, and the index “(t)” (for instance, “P5 (t)”, denotes patient 5, tumor sample), while the data for the peri-tumoral (reference) samples are represented by solid symbols and the index “(r)”. The data sets also include the spin–lattice relaxation rates at 18.7 MHz. The spin–lattice relaxation process has turned out to be single exponential—the corresponding magnetization curves (^1^H magnetization versus time) are presented in the Supplementary Materials, Figures S1 and S2.

The ^1^H spin–lattice relaxation rates, $R_{1}(\omega )$, (ω denotes the resonance frequency in angular frequency units), can be reproduced in the whole frequency range in terms of a power-law function:

(1) $$R_{1}\left(\omega \right)\;=\;C\omega^{-\alpha }$$

where α denotes the power-law factor, while C is a phenomenological pre-factor (in $s^{\left(\alpha -1\right)}$). The obtained values of α are collected in Table 2.

The power-law factor, The power-law factor, α, ranges between 0.27 and 0.33. This narrow range of the variations implies that there is no statistically relevant difference between the averaged α values for the pathological and reference tissues. One should, however, note that α = 0.3 can be considered as a characteristic relaxation property for lung tissues.

**Figure 2:**
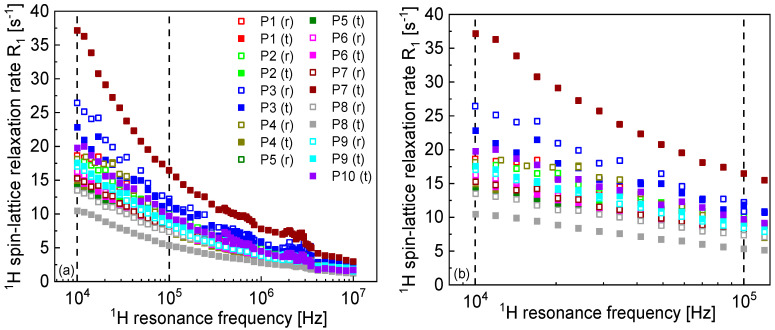
(**a**) Comparison of ^1^H spin–lattice relaxation rates already shown in Figure 1. Vertical lines mark the frequency range from 10 kHz to 100 kHz. (**b**) Decreasing of the ^1^H spin–lattice relaxation rates in the indicated frequency range.

Figure 2a includes a comparison of the ^1^H spin–lattice relaxation rates obtained for all cases. The relaxation rates converge at higher frequencies, showing significant differences in the low frequency range. Therefore, it is of interest to discuss relative changes in the spin–lattice relaxation rates in a specified frequency range, from $\nu_{1}$ to $\nu_{2}$, focusing on low frequencies. The relative change (decreasing) of the ^1^H spin–lattice relaxation rates is given by a parameter $\xi \;=\;\frac{R_{1}\left(\nu_{1}\right)-R_{1}\left(v_{2}\right)}{R_{1}\left(\nu_{1}\right)}$. As far as the frequencies are concerned, $\nu_{1}\;$= 10 kHz and $\nu_{2}\;$= 100 kHz have been chosen to cover one order of magnitude in the low frequency range (Figure 2b).

**Figure 3:**
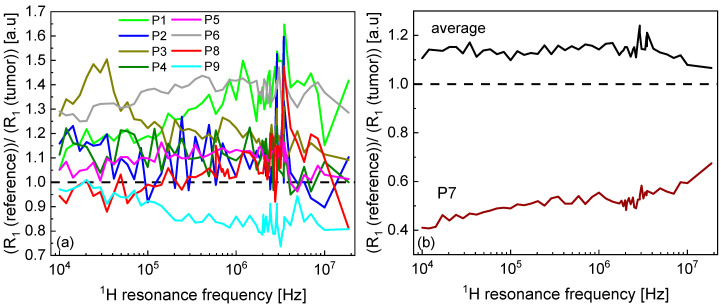
(**a**) Ratio between ^1^H spin–lattice relaxation rates for reference and pathological tissues for P1–P6, P8 and P9. (**b**) Averaged value of the ratios shown in (**a**) and the ratio for P7.

The ξ parameter ranges between 43% and 58%—the value is determined by the power-law factor, α. For α of about 0.3, the relaxation rates decrease by about a factor of 2 (50%). It is of interest to inspect the ratio between the ^1^H spin–lattice relaxation rates for the reference and pathological tissues in the covered frequency range. The ratios for the individual cases are presented in Figure 3. A closer inspection of Figure 1 shows that in the majority of the cases, the ^1^H spin–lattice relaxation rates for the reference tissues are higher than for the corresponding pathological samples. The relationship is different for P7 (adenocarcinoma)—in this case, the relaxation is much faster for the pathological tissue, and (to some extent) for P9 (pleomorphic lung cancer). In the last case, this effect is, however, much less pronounced. Figure 3a shows the ratio between the relaxation rates for the reference and the pathological tissues for all cases except for P7. The average ratio (Figure 3b) is, in a good approximation, frequency independent (except for the frequency range from about 2 MHz to 4 MHz in which Quadrupole Relaxation Enhancement (QRE) effects are visible), being about 1.15. The ratio for P7 is much different (Figure 3b).

The QRE effects, which slightly manifest themselves in the spin–lattice relaxation data, stem from ^1^H-^14^N dipole–dipole interactions. ^14^N nuclei possess quadrupole moments (as a result of the spin quantum number 1) and, consequently, their energy level structure is dominated (in the covered frequency range) by the quadrupole coupling. At some magnetic fields, the ^1^H resonance frequency matches one of the transition frequencies of ^14^N, and then the ^1^H magnetization can be taken over by ^14^N nuclei (under specific conditions, including sufficiently slow time fluctuations of the ^1^H-^14^N dipole–dipole interactions), leading to an effective, frequency-specific enhancement of the ^1^H spin–lattice relaxation. QRE effects (especially the frequency position of the relaxation maxima, often referred to as “quadrupole peaks”) are a very sensitive fingerprint of changes in the molecular arrangement (by affecting the electric field gradient at the ^14^N site) [13,31,32]. In this case, they are only weakly pronounced, likely being obscured by the ^1^H-^1^H relaxation contribution. The QRE effects imply the presence of a fraction of hydrogen atoms that are involved in a slow dynamical process (even immobilized), and the frequency position of the quadrupole peaks (this term is often used in the literature) is determined by the value of the electric field gradient tensor at the ^14^N site. As the effects are weakly pronounced, their quantitative analysis is hardly possible. However, a closer inspection of Figure 1 and Figure 2 (especially the cases of P1, P7 and P10) indicates two groups of quadrupole peaks, suggesting that there are two fractions of ^14^N nuclei (belonging to different macromolecular compounds present in tissues or different functional groups) undergoing a slow motion.

**Figure 4:**
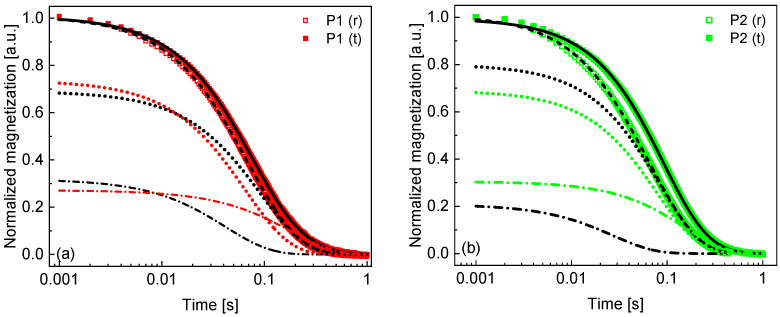

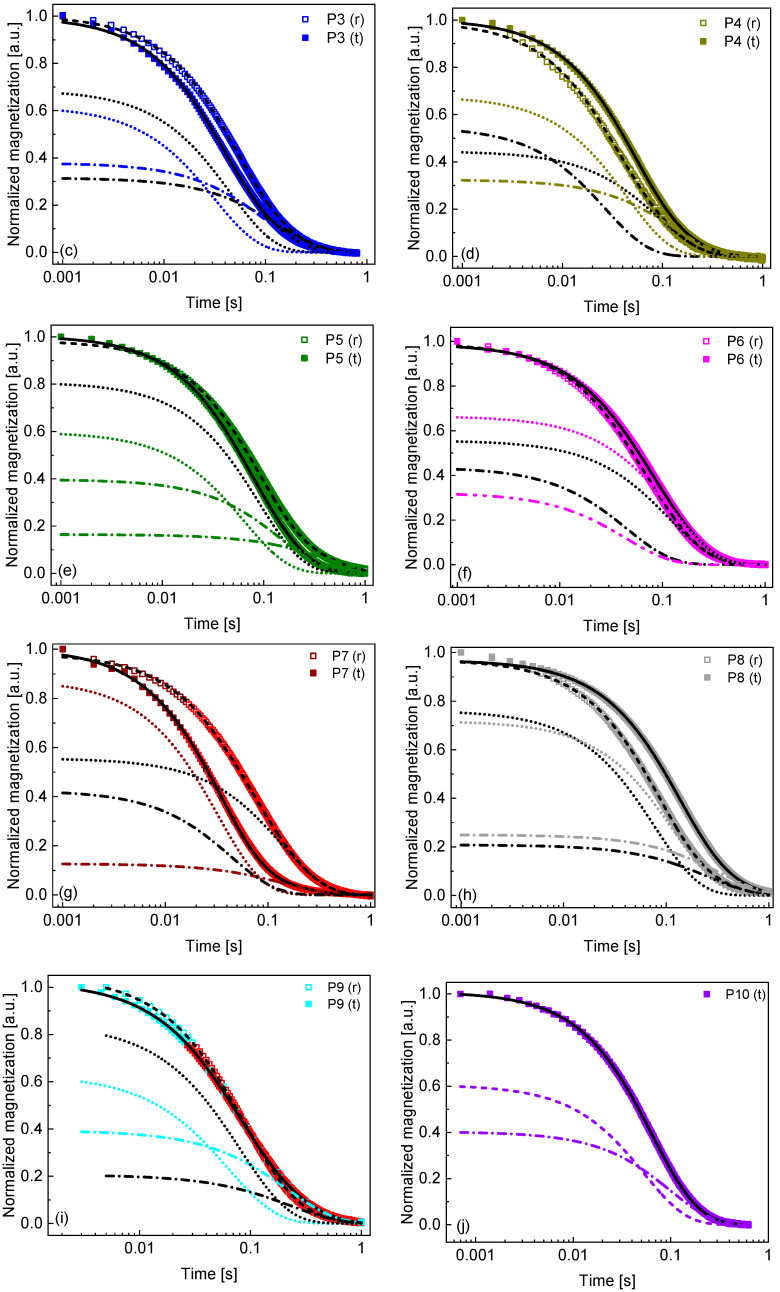
(**a**–**j**) ^1^H magnetization versus time in spin–spin relaxation experiments at 18.7 MHz for lung tissues. Solid lines—biexponential fits decomposed into two contributions: $R_{2,slow}$—black dotted lines for the reference tissue and color dotted lines for tumors, $R_{2,fast}$—black dashed dotted lines for the reference tissue and color dashed dotted lines for tumors (R-square values for the fits range between 0.998 and 0.999).

The ^1^H spin–lattice relaxation measurements at 18.7 MHz have been complemented by spin–spin relaxation experiments at the same frequency. The spin–spin relaxation process has turned out to be bi-exponential in all cases. Consequently, the time evolution of the ^1^H magnetization obtained in the spin–spin relaxation experiment has been reproduced using the following expression:

(2) $$M\left(t\right)\;={\;A}_{slow}exp\left(-R_{2,slow}t\right)+A_{fast}exp\left(-R_{2,fast}t\right)$$

where $R_{2,slow}$ and $R_{2,fast}$ denote the spin–spin relaxation rates associated with the slower and the faster relaxation contributions, respectively, while $A_{slow}$ and $A_{fast}$ are the corresponding amplitudes. The data are shown in Figure 4 for the individual patients.

## 3. Discussion

Beginning the discussion with the ^1^H spin–lattice relaxation effects, the first observation to be made is the power-law dependence of the relaxation rates on the resonance frequency. The power-law factor, α, varies in a narrow range, between 0.27 and 0.33, with the average value of about 0.3. Power-law dependencies of spin–lattice relaxation rates on the resonance frequency have been observed for systems including macromolecular fractions (such as proteins or polymers) and water, and are attributed to the dynamics of the macromolecular backbones reflected by the motion of the bound water molecules. For water solutions (mixtures) of proteins, the power-law factor typically ranges between 0.5 and 0.8 [33,34,35,36,37], although a power-law factor of about 0.94 was reported for water–collagen mixtures including about 40% wt. of collagen [38]. It is worth mentioning that for protein solutions, Lorentzian forms of spectral density functions characteristic of rotation dynamics evolve into power-law forms with increasing protein concentration, as a result of cross-linking leading to their immobilization. As far as polymer systems are concerned, several characteristic power-law regimes have been theoretically predicted (and to some extent, experimentally confirmed). The lowest power-law factor of 0.25 is predicted for the inter-molecular relaxation contribution for the Rouse and reptation dynamics of polymers, then 0.5 for the intra-molecular relaxation contribution in the case of the reptation motion, up to 0.625 (5/8) and 0.75 for the intra-molecular and inter-molecular relaxation contributions, respectively, for constrained Rouse dynamics [39,40,41]. In comparison, the power-law factor determined for lung tissues is low, and this finding is worth attention. Moreover, to interpret spin–lattice relaxation data for protein and polymer systems over a broad range of frequencies, one needs to include (in the majority of cases) other relaxation contributions—the power-law term is only one of them. For lung tissue, we are able to reproduce the relaxation data entirely in terms of the power-law function. The comparison of the power-law factors shows that this quantity varies in a broad range and reflects different kinds of motion (such as backbone vibrations in proteins or the reptation dynamics of polymers). The common denominator of the power-law dependencies of the relaxation rates on the resonance frequency is, however, a highly restricted dynamic. We prefer not to speculate on this subject, although one might think about the dynamics of strongly bound water molecules reflecting the collective dynamics of the macromolecular fraction, as the reason for this effect.

The pathological tissues have been identified as different kinds of tumors, according to Table 1. The natural question in this context is whether one can distinguish between pathological and reference tissues using the ^1^H spin–lattice relaxation data. The straightforward answer, based on the cases shown in this work, is no. At the same time, some of the observed effects indicate the diagnostic potential of this approach and should be further explored. One of them is the ratio between the relaxation rates for the reference and the tumor tissues. Excluding the case of P7 (adenocarcinoma), the ratio between the relaxation rates is larger than 1 (the relaxation process for the reference tissue is faster), with pleomorphic lung cancer (P9) showing the opposite effect. It is worth noting that when excluding the P9 case, the ratio between the relaxation rates for the reference and the pathological tissues increases, reaching about 1.2—this can be considered as a step towards diagnostic applications. At the same time, the noticeable decrease in the spin–lattice relaxation rates for pleomorphic lung cancer (compared to the reference tissue) also holds diagnostic promises. Of course, one should not draw any conclusions on the basis of a single case. Following this line, there are two cases of adenocarcinoma: P3 and P7. For P7, the ^1^H spin–lattice relaxation rates are much lower for the tumor tissue than for the reference one. For P3, the relationship is inverse. However, in both cases, the differences in the relaxation rates are significant, indicating considerable changes in the molecular dynamics and arrangement that are worth further exploration, as well as from a diagnostic perspective.

At 18.7 MHz, we have also performed ^1^H spin–spin relaxation experiments. The spin–spin relaxation process is bi-exponential in all cases. This implies the presence of two pools of hydrogen atoms contributing to the relaxation process. The analysis of the magnetisation evolution has led to two spin–spin relaxation rates, $R_{2,slow}$ and $R_{2,fast}$ and a ratio between the amplitudes of these two relaxation contributions: $r\;=\;\frac{A_{fast}}{A_{slow}}$. The $R_{2,slow}$ values range between about 4 s^−1^ and 12 s^−1^, and for $R_{2,fast}$, the span is from about 8 s^−1^ to 12 s^−1^, with the ratio between the relaxation rates, $\frac{R_{2,\;fast}}{R_{2,slow}}$, from about 1.9 to 4.9. One might attribute the slower spin–spin relaxation contribution to the fraction of water molecules bound to the macromolecules and then treat the single exponential spin–lattice relaxation process as its counterpart. The ratio $\frac{R_{2,\;slow}}{R_{1}}$ ranges between 1.7 and 6.1 (fulfilling the condition originating from the quantum-mechanical framework of spin relaxation process that spin–spin relaxation rates are never lower than the corresponding spin–lattice relaxation rates). Then, the spin–spin relaxation process described by $R_{2,\;fast}$ could be attributed to hydrogen atoms belonging to the macromolecular fraction of tissues. A faster spin–spin relaxation process implies a slower molecular motion (assuming that dipolar relaxation constants are comparable), and one can expect a slower dynamic of the macromolecules compared to the motion of the bound water molecules. One should, however, be aware that this attribution remains a hypothesis. The parameter $r\;=\;\frac{A_{fast}}{A_{slow}}$ is always lower than 1 (or 100 when expressed in %)—this means that the amplitude of the faster spin–spin relaxation contribution is always lower than the slower one, although the ratio shows a large spread. As far as the lower spin–spin relaxation rate, $R_{2,\;slow}$, is concerned, its averaged value for the tumor tissues (6.3 ± 0.2) s^−1^ is lower than the corresponding averaged value for the reference tissue (7.7 ± 0.3) s^−1^, and the difference exceeds the uncertainty range. Discussing this ratio, it is worth noticing that freezing can change the relative fractions of water molecules of slow and fast mobility due to lysis.

The presented studies give insight into relaxation properties and, hence, molecular dynamics and organization of tissues. This subject is rarely addressed in the literature (outside of the context of Magnetic Resonance Imaging, focusing on a single, high resonance frequency). Tissues are undoubtedly complex, heterogeneous molecular systems, and frequency-dependent relaxation experiments are a source of information about the molecular motion and organization that cannot be obtained by other methods. The presented examples reveal several characteristic relaxation features of lung tissues, also showing effects that are worth further exploring in the context of diagnostics.

## 4. Materials and Methods

^1^H spin–lattice relaxation experiments covering the frequency range from 10 kHz to 10 MHz were performed using a Fast Field Cycling relaxometer produced by STELAR (Mede, Italy). The studies were complemented by ^1^H spin–lattice and spin–spin relaxation experiments performed at 18.7 MHz using the equipment produced by Resonance Systems (Kirchheim unter Teck, Germany), often referred to as “Time-Domain relaxometer”. The experiments were conducted at 37 °C, with an accuracy of 1 °C. Pre-polarization was applied for fields below 4 MHz. The spin–lattice relaxation rates were obtained as a result of mono-exponential fits of magnetisation curves (^1^H magnetisation versus time). Examples of the magnetisation curves are given in the Supplementary Materials (Figures S1 and S2). The spin–spin relaxation experiments at 18.7 MHz were performed using the Carr–Purcell–Meiboom–Gill (CPMG) sequence [42] with 1000 echoes and 200 scans, while for the corresponding spin–lattice experiments, the Saturation Recovery sequence [43] was applied with the observation time of 4 s and 6 scans. The Supplementary Materials (Figure S3) include the time evolution of the ^1^H magnetization recorded in the spin–lattice relaxation experiments at 18.7 MHz, reproduced in terms of a single-exponential function.

## 5. Conclusions

The analysis of the ^1^H spin–lattice relaxation data shows that independently of the state of lung tissues (reference, different kinds of tumor), the frequency dependence of the relaxation rates on the resonance frequency follows (in the frequency range from 10 kHz to 18.7 MHz) a power-law function with a factor of about 0.3. The spin–lattice relaxation process is single exponential for all cases in the whole frequency range, and it can be attributed to the fraction of bound water molecules reflecting the dynamics of the macromolecular backbones. At the same time, the spin–spin relaxation process (at 18.7 MHz) has turned out to be bi-exponential in all cases. The spin–lattice relaxation rates characterizing the slower relaxation component range between about 4 s^−1^ and 12 s^−1^, while for the faster component, the relaxation rates range between about 11 s^−1^ and 37 s^−1^, with the ratio between the two relaxation rates in the range from 1.9 to 4.9. The amplitude of the faster relaxation contribution is always lower. The slower relaxation component can be treated as a counterpart of the spin–lattice relaxation process, while the faster one can be attributed to the macromolecular fraction of lung tissue. The spin–spin relaxation rates (for both relaxation contributions) are somewhat smaller (outside the uncertainty range) for tumor tissues. As far as the spin–lattice relaxation is concerned, the most pronounced differences between the relaxation rates for the reference and the pathological tissues are observed for adenocarcinoma and pleomorphic lung cancer. Although some of the presented results indicate a diagnostic potential of low-field relaxation data for lung tissues, at this stage, a diagnostic value should not be attributed to the parameters obtained from the quantitative analysis of the data.

## Figures and Tables

**Table 1:** Histopathological characterization of the tumors. “SUV” stems from Standardized Uptake Value (PET tomography), and it is a measure of the fraction of the injected radiotracer accumulated in a specific part of the body. SUV is measured only for areas with high metabolism or for regions that appear suspicious for tumors.

Case	SUV	Tumor Size (mm × mm)	Description
P1	2.9	2.8 × 2.8	Organized hematoma
P2	17.3	84 × 30	Squamous cell carcinoma
P3	25.0	48 × 35	Squamous cell carcinoma
P4	19.9	56 × 46	Adenocarcinoma
P5	2.0	25 × 29	Benign tumor from epithelioid cells
P6	9.6	80 × 90	Squamous cell carcinoma
P7	14.2	84 × 57	Adenocarcinoma
P8	12.2	65 × 51	Squamous cell carcinoma
P9	26.7	53 × 50	Pleomorphic lung cancer
P10	12.0	50 × 43	Squamous cell carcinoma

**Table 2:** Parameters characterizing ^1^H spin–lattice and spin–spin relaxation for lung tissues. The parameter ξ defined as $\xi =\frac{R_{1}\left(\nu_{1}\right)-R_{1}\left(v_{2}\right)}{R_{1}\left(\nu_{1}\right)}$, where $\nu_{1}$ = 10 kHz, $\nu_{2}$ = 100 kHz, while $r=\frac{A_{fast}}{A_{slow}}$; $R_{1}$ denotes the values of the spin–lattice relaxation rate at 18.7 MHz, where the obtained spin–spin relaxation rates at 18.7 MHz are denoted by $R_{2,slow}$ and $R_{2,fast}$. The uncertainties of the average values are represented by stand. dev.

Case	α	ξ [%]	$R_{2,slow}\;[s^{-1}]$	$R_{2,fast}\;[s^{-1}]$	r [%]	$\frac{R_{2,fast}}{R_{2,slow}}$	$\frac{R_{2,slow}}{R_{1}}$
P1 (r)	0.30 ± 0.01	48	10.8 ± 0.1	24.1 ± 0.77	46	2.2	6.4
P1 (t)	0.33 ± 0.01	54	5.6 ± 0.2	15.3 ± 0.20	36	2.7	2.7
P2 (r)	0.28 ± 0.01	43	11.99 ± 0.04	37.1 ± 0.89	26	3.1	6.1
P2 (t)	0.32 ± 0.01	55	6.4 ± 0.1	12.4 ± 0.11	38	1.9	4.6
P3 (r)	0.33 ± 0.01	59	7.5 ± 0.1	22.26 ± 0.04	46	3.0	3.2
P3 (t)	0.31 ± 0.01	53	10.11 ± 0.08	31.4 ± 0.3	61	3.2	4.7
P4 (r)	0.33 ± 0.01	50	10.9 ± 0.1	37.3 ± 0.7	81	3.4	7.0
P4 (t)	0.30 ± 0.01	51	7.62 ± 0.06	22.5 ± 0.2	48	2.9	5.4
P5 (r)	0.29 ± 0.01	44	6.72 ± 0.05	15.5 ± 0.2	66	2.3	4.9
P5 (t)	0.29 ± 0.01	49	2.37 ± 0.02	11.04 ± 0.04	20	4.7	1.7
P6 (r)	0.29 ± 0.01	52	6.61 ± 0.08	16.1 ± 0.1	36	2.4	4.4
P6 (t)	0.30 ± 0.01	53	7.49 ± 0.03	18.2 ± 0.2	76	2.4	5.1
P7 (r)	0.28 ± 0.01	49	6.68 ± 0.04	18.8 ± 0.2	88	2.8	4.5
P7 (t)	0.33 ± 0.01	58	5.91 ± 0.06	28.8 ± 0.1	19	4.9	2.7
P8 (r)	0.27 ± 0.01	47	3.91 ± 0.04	12.8 ± 0.1	27	3.3	2.9
P8 (t)	0.29 ± 0.01	51	2.95 ± 0.02	8.40 ± 0.04	35	2.9	2.8
P9 (r)	0.33 ± 0.01	51	4.18 ± 0.04	11.89 ± 0.05	21	2.8	3.4
P9 (t)	0.30 ± 0.01	48	4.38 ± 0.03	14.6 ± 0.1	34	3.3	2.9
P10 (t)	0.33 ± 0.01	52	10.1 ± 0.1	18.8 ± 0.2	66	1.9	5.2
Avg. (t)	0.31 ± 0.02	52 ± 3	6.3 ± 0.2	18.2 ± 0.7	43 ± 19	3.2 ± 0.9	3.7 ± 1.3
Avg. (r)	0.30 ± 0.02	49 ± 4	7.7 ± 0.3	21.8 ± 0.9	49 ± 24	2.8 ± 0.4	4.8 ± 1.4

## Data Availability

The original data presented in the study are openly available in zenodo at https://doi.org/10.5281/zenodo.16881450.

## Supplementary Materials

The following supporting information can be downloaded at: https://www.mdpi.com/article/10.3390/molecules30194002/s1, Figure S1: Examples of ^1^H magnetization versus time in the FFC-NMR spin-lattice experiments (P1 (t)–P10 (t)). Solid lines—single–exponential fits. The top time axis corresponds to the frequencies of 10 kHz, 100 kHz and 1 MHz, while the bottom one to 10 MHz; Figure S2: Examples of ^1^H magnetization versus time in the FFC-NMR spin-lattice experiments (P1 (r)–P9 (r)). Solid lines—single–exponential fits. The top time axis corresponds to the frequencies of 10 kHz, 100 kHz and 1 MHz, while the bottom one to 10 MHz; Figure S3: ^1^H magnetization evolution in spin–lattice relaxation TD-NMR experiments. Solid lines—single exponential fits.
