# Supplementary material for: 1H NMR Relaxation Processes in Lung Tissues at Low Magnetic Fields
Source: Molecules. 2025 Oct 7;30(19):4002. doi: 10.3390/molecules30194002 (PMC12525575; doi:10.3390/molecules30194002)
Supplement: Supplementary file 1 [file molecules-30-04002-s001.zip › molecules-3811842-supplementary.pdf]

Supplementary Material

1H NMR Relaxation Processes in Lung Tissues at Low Magnetic Fields

Karol Kołodziejski [1], Farman Ullah [1], Łukasz Klepacki [2], Jerzy Gielecki [2] and Danuta Kruk [1,*]

[1] Department of Physics and Biophysics, University of Warmia and Mazury in Olsztyn, Michała Oczapowskiego 4, 10-719 Olsztyn, Poland; karol.kolodziejski@uwm.edu.pl (K.K.); farman.ullah@uwm.edu.pl (F.U.)

[2] Department of Anatomy and Histology, Faculty of Medicine, Collegium Medicum, University of Warmia and Mazury in Olsztyn, 10-082 Olsztyn, Poland; lukasz.klepacki@uwm.edu.pl (Ł.K.); jerzy.gielecki@uwm.edu.pl (J.G.)

* Correspondence: danuta.kruk@uwm.edu.pl

Figure S1. and **Figure S2.** show examples of ^{1}H magnetization versus time in FFC-NMR spin-lattice relaxation experiments.

Figure S3. Show ^{1}H magnetization evolution in spin – lattice relaxation TD-NMR experiments.

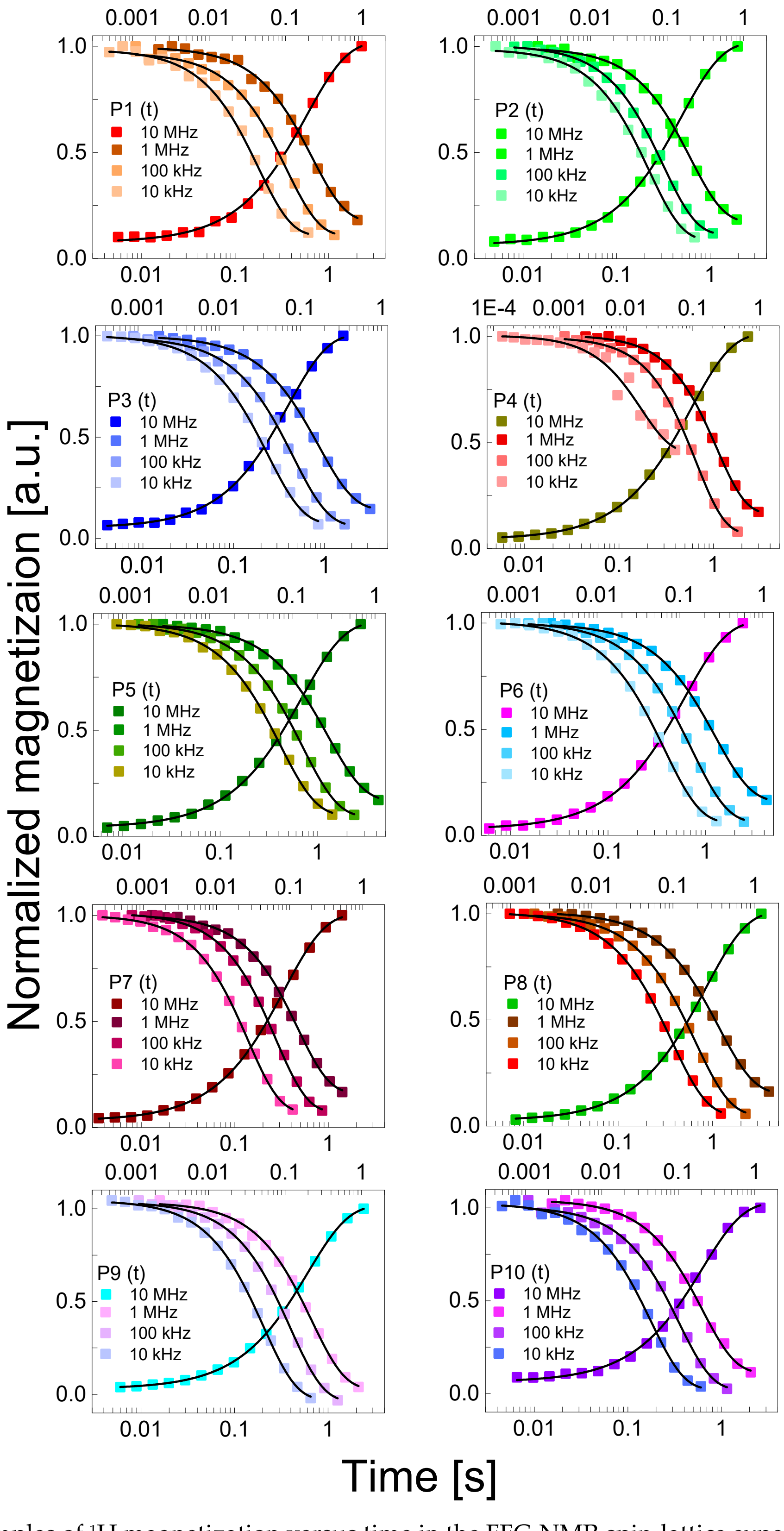

Figure S1. Examples of ^{1}H magnetization versus time in the FFC-NMR spin-lattice experiments (P1(t) – P10(t)). Solid lines – single – exponential fits. . The top time axis corresponds to the frequencies of 10kHz, 100kHz and 1MHz, while the bottom one to 10MHz.

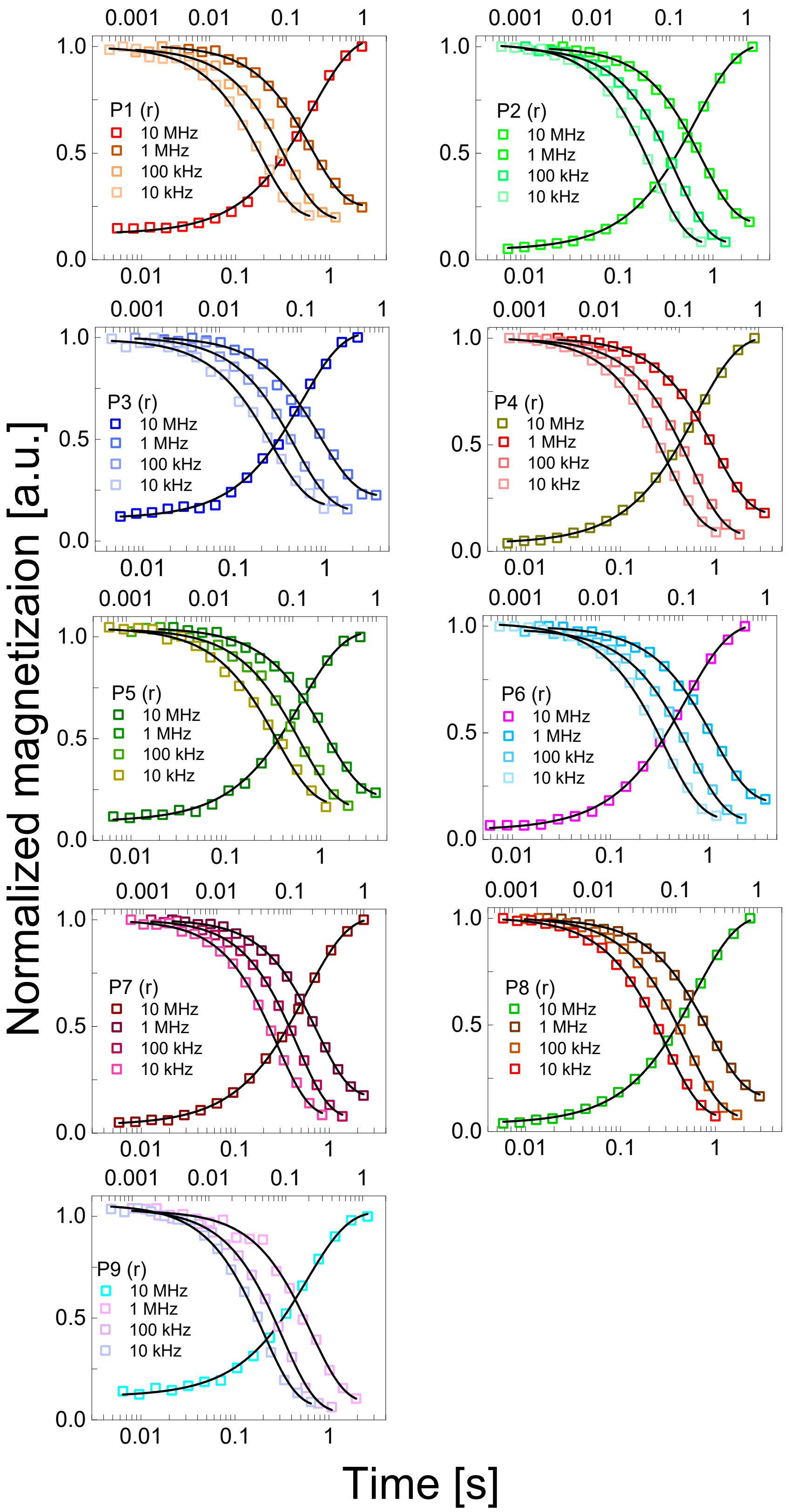

Figure S2. Examples of ^{1}H magnetization versus time in the FFC-NMR spin-lattice experiments (P1(r) – P9(r)). Solid lines – single – exponential fits. The top time axis corresponds to the frequencies of 10kHz, 100kHz and 1MHz, while the bottom one to 10MHz.

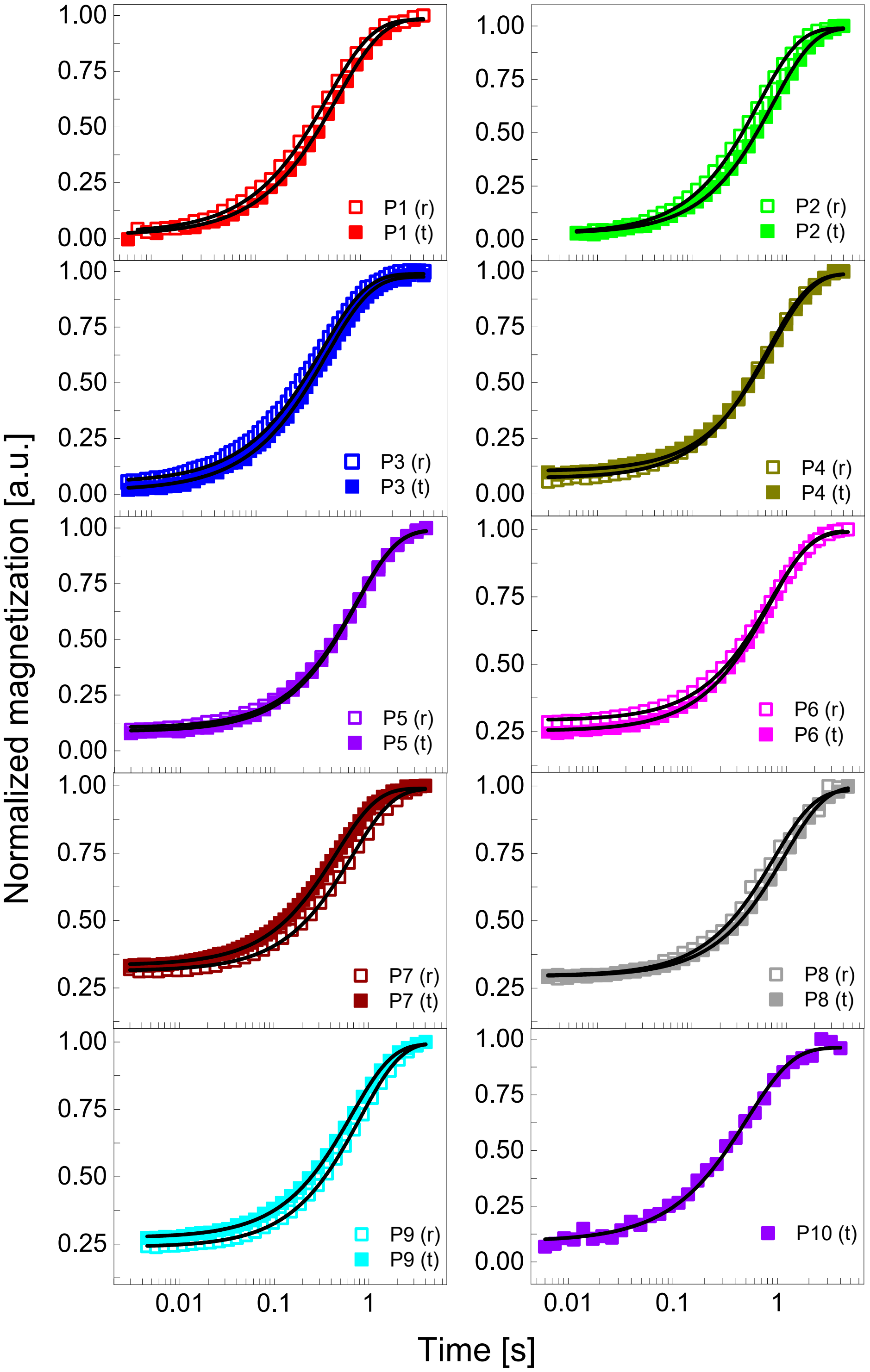

Figure S3. ^{1}H magnetization evolution in spin – lattice relaxation TD-NMR experiments. Solid lines – single exponential fits.
